# Mediators of liver inflammation and carcinogenesis

**DOI:** 10.1007/s00281-021-00880-x

**Published:** 2021-09-16

**Authors:** Johannes Herkel, Dirk Schmidt-Arras

**Affiliations:** 1 Department of Medicine I, University Medical Center Hamburg-Eppendorf, Martinistr. 52, 20246 Hamburg, Germany; 2 Institute of Biochemistry, Christian-Albrechts-University Kiel, Rudolf-Höber-Str. 1, 24118 Kiel, Germany

The present issue of *Seminars in Immunopathology* covers a number of reviews that summarize emerging concepts, potential therapeutic targets and mechanisms that underly the immunopathology of liver inflammation and liver cancer. Liver inflammation is one of the most common medical conditions in the world, which can be caused by various insults, including viral infection, toxins, hereditary metabolic diseases or autoimmune diseases. Frequently, chronic liver inflammation results in liver cirrhosis or liver cancer, both being leading causes of death worldwide. In fact, liver cirrhosis has caused more than 1.32 million deaths in the year 2017 [1]. In 2020, more than 900,000 new cases of liver cancer have been diagnosed, and more than 800,000 people have died from liver cancer, making it the sixth most common tumour worldwide with the third highest mortality rate [2]. The majority of these liver cancers develop from hepatocytes, the liver parenchymal cells (hepatocellular carcinoma) or from cholangiocytes, the cells lining the bile ducts (cholangiocarcinoma). Irrespective of the underlying cause, liver carcinogenesis most often progresses along a sequence of chronic inflammatory liver damage, compensatory regeneration and fibrotic and cirrhotic tissue remodelling [3]. Thus, there is urgent, yet unmet need for curative treatments tackling liver inflammation and carcinogenesis. However, only a deep understanding of the immunopathology will enable the development of novel, effective therapies. This collection of reviews seeks to wrap up several key issues in the immunopathology of liver inflammation and liver cancer.

## Homoeostatic inflammation vs. pathogenic tissue remodelling

It is important to understand that the maintenance of a healthy liver probably involves some degree of homoeostatic liver inflammation that facilitates the removal of damaged or aberrant cells. This self-limited homoeostatic tissue maintenance produces only short episodes of inflammation and fibrosis which both resolve after removal of the triggering event. In contrast, liver diseases develop from persistent liver inflammation and chronic fibrosis, leading to cirrhosis and cancer. The reasons for chronic persistence of inflammation and aberrant forms of tissue regeneration are not always clear. But inflammation is always a trade-off between inflammatory tissue damage that facilitates the removal of aberrant or infected cells, and preservation of vital tissue function. Possible causes of persisting inflammation might thus include the inability to remove the triggering event, such as a pathogen. Indeed many pathogens have evolved successful strategies to evade the immune response in the liver. Alternatively, chronic liver inflammation and aberrant tissue regeneration might be caused by imbalanced immune responses, resulting from misinterpretation of the numerous, partly conflicting signals immune cells are exposed to.

The review by Bosurgi and Rothlin in this issue addresses the management of cell death in the context of parasitic infections [4]. The authors show that the recognition and phagocytosis of dead cells are of a major relevance for shaping the immune response. However, they also show that parasites have developed strategies to interfere with and make use of phagocytosis signals. Moreover, the relevance for liver diseases and potential therapeutic targets are discussed.

Monocytes are a major cell type involved in liver inflammation and the response to liver infection. The review by Sellau et al. in this issue [5] discusses the extraordinary plasticity of this cell population upon recruitment to the liver in the context of liver infections. The authors argue that the plastic response of monocyte-derived cells to infection can result in pathogen clearance, but sometimes also in excessive fibrosis or immunopathology. Potential treatment strategies are discussed.

Rangaswamy et al. in this issue explore the underestimated role of the contact system in liver injury and inflammation [6]. Coagulation and vascular permeability can potentially have a major impact on liver inflammation and liver diseases. Moreover, as most coagulation factors are produced in the liver, impaired liver function in liver diseases can result in bleeding disorders. The authors also discuss potential therapeutic strategies.

The role of NK cells in liver inflammation is discussed in the review by Highton et al. in this issue [7]. NK cells are of particular importance for tissue maintenance, as they can detect and kill infected or aberrant cells. However, too much killing can contribute to chronic liver injury and liver disease. Recent discoveries of distinct NK subsets suggest heterogeneous NK cell functions. The authors discuss potential contributions of NK cells to specific liver diseases and potential treatment strategies.

Infections with hepatitis viruses are a major trigger of chronic liver inflammation and liver disease. In this issue, Dandri et al. discuss the innate immune response in viral hepatitis, its consequences for T cell recognition and how this impacts on viral persistence or clearance [8]. The authors elucidate viral strategies to manipulate and shape the immune response and therapeutic strategies that might specifically target viral evasion.

The review of Muscate and Woestemeier et al. in this issue [9] highlights the heterogeneity of CD4 T cell responses in liver diseases, which is related to the plasticity of T cells in response to the hepatic microenvironment. The authors investigate the relative contributions of CD4 T cell subsets and differentiation stages to liver inflammation and disease.

## Extrinsic regulation of liver inflammation

Accumulating evidence suggests that the gut microbiome can exert an influence on the regulation of immunity in other organs. As the liver receives the major part of its blood supply through the portal vein from the gut, it is extensively exposed to gut-derived signals. Two reviews in this issue focus on two major pathways that can translate microbiome-derived signals into hepatic immune responses.

Carambia and Schuran [10] give an overview on the ligand-activated transcription factor AHR that can integrate diverse nutritional, microbial or environmental molecular signals into cellular transcriptional programs. They argue that AHR can influence hepatic immunity in many ways, both in healthy conditions and in liver inflammation. Moreover, therapeutic targeting of AHR in liver disease, fibrosis and cancer is discussed.

Evangelakos et al. explore the role of bile acids that are modified by gut bacteria and regulate hepatic immunity through diverse bile acid receptors [11]. Bile acid metabolism, interaction with the microbiome and role in immune regulation and liver disease are discussed. The authors argue that bile acids and their receptors are promising therapeutic targets particularly in cholestatic and metabolic liver diseases.

## The impact of inflammation on liver tumorigenesis

Activation of inflammatory cells, associated with the secretion of pro-inflammatory cytokines, is observed during hepatic infection but also upon parenchymal damage. As an acute response, cytokines are important mediators of pathogen defence and regenerative processes. As an example, the cytokine interleukin 6 (IL-6) was identified as a key regulator of both processes by (i) inducing acute-phase-proteins that dampen infection, and (ii) promoting hepatocyte proliferation upon acute damage [12].

However, persistent inflammation of the liver is considered to predispose to tumour formation by persistent promotion of parenchymal cell proliferation, but also the suppression of an appropriate anti-tumour immune response. Hepatocellular carcinoma (HCC) and cholangiocarcinoma (CCA) are the most common tumour types occurring in the liver and in particular HCC is considered a prime example of an inflammation-driven cancer. However, the decision whether a cytokine is pro-tumorigenic or tumour-suppressive does not only depend on the type of cytokine but also its concentration and the cell type it acts on. This has to be considered in therapeutic decision-making and for the design of novel therapeutic approaches.

The lymphocyte-derived cytokine IL-22 is a major regulator of tissue regeneration. Lücke et al. [13] provide an overview on the regulation of IL-22 in liver regeneration and carcinogenesis, which depends much on its endogenous antagonist, IL-22BP. The authors elucidate the contributions of the IL-22/IL-22BP system to various liver diseases, fibrosis and cancer. They argue that dysregulated IL-22 activity can promote both liver cirrhosis and liver cancer, due to the high potency of IL-22 to promote regeneration. Therapeutic options are discussed.

The IL-6 cytokine family, which signals through the gp130–STAT signalling axis, is of central relevance for inflammation and carcinogenesis. Schmidt-Arras et al. in this issue [14] dissect the complex role of this cytokine family in liver inflammation and liver carcinogenesis, which has long been thought to primarily be tumour-promoting. The authors argue that gp130 signalling can also have tumour-preventing effects, mainly by regulating senescence. The authors discuss therapeutic options taking both the tumour-promoting and tumour-preventing effects of gp130 signalling into account.

## Conclusion/Perspective

This issue cannot address all aspects of liver inflammation. However, the collection of reviews in this issue highlights several key issues that are emerging as essential regulators in the regulation of liver inflammation and its progression towards liver cirrhosis and liver cancer. Importantly, these reviews not only discuss the immunopathogenesis of these conditions but also propose research priorities, and, most notably, suggest targets for therapeutic intervention. Therefore, the current collection functions as a roadmap that can guide our way towards effective and specific treatments for serious clinical conditions associated with liver inflammation, cirrhosis and cancer.
